# Is the dose distribution distorted in IMRT and RapidArc treatment when patient plans are swapped across beam‐matched machines?

**DOI:** 10.1120/jacmp.v17i5.6104

**Published:** 2016-09-08

**Authors:** Chockkalingam Krishnappan, Chandrasekaran Anu Radha, Vendhan Subramani, Madhan Kumar Gunasekaran

**Affiliations:** ^1^ Department of Medical Physics Apollo CBCC Hospitals Gandhinagar Gujarat India; ^2^ School of Advanced Sciences, VIT University Vellore Tamilnadu India

**Keywords:** beam‐matched linacs, beam‐matching criteria, dosimetric evaluation, TPS commissioning

## Abstract

The purpose of this study is to evaluate the degree of dose distribution distortion in advanced treatments like IMRT and RapidArc when patient plans are swapped across dosimetrically equivalent so‐called “beam‐matched” machines. For this purpose the entire work is divided into two stages. At forefront stage all basic beam properties of 6 MV X‐rays like PDD, profiles, output factors, TPR20/10 and MLC transmission of two beam‐matched machines — Varian Clinac iX and Varian 600 C/D Unique — are compared and evaluated for differences. At second stage 40 IMRT and RapidArc patient plans from the pool of head and neck (H&N) and pelvis sites are selected for the study. The plans are swapped across the machines for dose recalculation and the DVHs of target and critical organs are evaluated for dose differences. Following this, the accuracy of the beam‐matching at the TPS level for treatments like IMRT and RapidArc are compared. On PDD, profile (central 80%) and output factor comparison between the two machines, a maximum percentage disagreement value of −2.39%,−2.0% and −2.78%, respectively, has been observed. The maximum dose difference observed at volumes in IMRT and RapidArc treatments for H&N dose prescription of 69.3 Gy/33 fractions is 0.88 Gy and 0.82 Gy, respectively. Similarly, for pelvis, with a dose prescription of 50 Gy/25 fractions, a maximum dose difference of 0.55 Gy and 0.53 Gy is observed at volumes in IMRT and RapidArc treatments, respectively. Overall results of the swapped plans between two machines' 6 MV X‐rays are well within the limits of accepted clinical tolerance.

PACS number(s): 87.56.bd

## I. INTRODUCTION

Beam matching is the concept of altering or tuning the beams of teletherapy machines so that they match with one another. Beam commissioning in conventional Co‐60 machines uses a single set of universal beam profile charts supplied by the manufacturer. These charts resemble the Co‐60 beam of all machines and thus the concept of beam matching was irrelevant. However, with time Co‐60 units have almost been completely replaced by medical linear accelerators. Computer‐controlled linear accelerators (linacs) that generate high‐energy X‐rays havetheir own specific and unique beam characteristics. Hence, each beam from a linac has to be commissioned individually^(1)^ before it is used clinically for patients.

Steep increase in cancer incidence has made radiotherapy centers think about having a second linac in their department. Often, a second linac is considered as standby machine if one of them is inevitably down. So, the two linacs are made dosimetrically equivalent through so‐called beam‐matching.^(2)^ The term “beam‐matched linacs” ensures that the X‐ray beams of matched linacs exhibit almost similar dosimetric characteristics. One of the clear advantages of beam‐matching linear accelerators is the improved efficiency and flexibility in patient treatment for institutions with two or more linear accelerators. Effects of beam‐matching results and beam data reproducibility for various accelerators have previously been analyzed and presented by several authors.^2^, ^3^, ^4^, ^5^, ^6^, ^7^

The beam‐matching criteria basically depend on depth dose/ionization curves, as well as beam profiles measured in both inline and crossline directions under vendor‐defined prescribed geometry.^(8)^ Even though during the accelerators' customer acceptance procedure all the vendor‐defined criteria are duly fulfilled, they are inadequate for beam matching. The vendor‐defined criteria consider only some points on profiles and depth dose curves instead of the full portion of the same. Apart from this they do not include the output factors and therefore run a risk of good agreement solely due to normalization. All vendor‐defined measurements are carried only for open static fields with no inclusion of multileaf collimators (MLC), whereas MLC is an integral part of modern‐day radiotherapy. MLC effects on beam‐matched linacs can bring about a severe alteration in patient dose distributions, especially in inverse planning–based advanced treatments like intensity‐modulated radiation therapy (IMRT) and RapidArc (Varian Medical Systems, Palo Alto, CA). Even though the beam matching is done at the factory, its accuracy has to be ensured in clinics before shifting the patients across the machines if the same dose distributions are to be achieved for the patient. Although several studies have been carried out on beam data comparisons of beam‐matched linacs, there are no data available on the effects of beam‐matching at the patients' levels, especially in high‐end treatments like IMRT and RapidArc. The aim of this study is to evaluate the accuracy of beam matching by overcoming the shortfalls of vendor‐defined criteria and to study the effects of beam matching in advanced treatments like IMRT and RapidArc.

## II. MATERIALS AND METHODS

Recently at our center, we commissioned a Varian 600 C/D Unique linac. It is a magnetron‐based low‐energy linac capable of generating only one energy of X‐ray photons (6 MV). It has electron gun, standing waveguide, tungsten target and flattening filter all together as one complete central beam‐line unit. There are no bending magnets as the entire waveguide unit is mounted vertically above the ion chamber. This linac is equipped with the 120 Millennium Multi‐Leaf Collimator as tertiary MLC, in addition to beam‐limiting jaws (upper and lower). This linac has been beam‐matched with the already existing Varian Clinac iX Trilogy at our department. Other than collimating diaphragms and MLCs, the linac has a completely different head and waveguide design. Both the linacs are capable of delivering high dose rates (up to 600 MU/min). The accuracy of beam matching between the two linacs is evaluated in two segments.

### A. At machine commissioning

For both machines' 6 MV X‐rays, basic beam data like percentage depth doses (PDDs) and cross‐beam profiles are measured in a radiation field analyzer (Blue Phantom, IBA Dosimetry GmbH, Schwarzenbruck, Germany) using CC13 chambers (IBA Dosimetry) for open fields. As per TRS398 protocol^(9)^ the beam quality index (TPR 20/10) is measured for 6 MV X‐rays of both the machines. Open‐beam output factors, along with their individual scatter components (phantom and head) are also measured for both the machines. FC65 chamber (IBA Dosimetry) and 1D Phantom (IBA dosimetry) are used to measure TPR 20/10 and output factors. Dosimetric leaf gap (DLG) and transmission of multi‐leaf collimators (MLCs) of both the machines are measured using CC13 chamber placed in an RW3 phantom (IBA Dosimetry).

A comparison is made between the PDDs of 6 MV X‐rays from both the machines at all depths for different field sizes ranging from $4\times 4\;{\text{cm}}^{2}$ to $40\times 40\;{\text{cm}}^{2}$. Before analysis, the PDDs of the machines at each field size were normalized to 100% at $D_{\text{max}}$ of the Clinac iX. Disagreement in PDD (%) [(PDD (Clinac iX) – PDD (Unique))] is then calculated at all depths on that particular field size by simple subtraction of PDD values of both the machines.

Beam profiles in the cross plane of the both machines for different field sizes (3×3,4×4,6×6,10×10,12×12,15×15,20×20,25×25, and $30\times 30\;{\text{cm}}^{2}$) at five different depths ($D_{\text{max}}$, 5, 10, 20, and 30 cm) are compared. All profiles at all depths are normalized to 100% at central axis. Profile disagreement analysis between the machines is made by calculating the difference in profile values [(Clinac iX –Unique)] at different regions (central 80% and penumbra).

The difference between the TPR 20/10 values of 6 MV X‐rays of both the machines are calculated. With the Clinac iX machine as baseline reference, the deviation in overall output factors, as well as phantom and head scatter factors, for the Unique are calculated and compared at all field sizes from $3\times 3\;{\text{cm}}^{2}$ to $40\times 40\;{\text{cm}}^{2}$ Percentage difference in output factors and TPR20/10 is calculated as {[value (Unique)–value (Clinac iX)]/value (Clinac iX)}×100%.

The difference in MLC transmission and DLG values (Clinac iX –Unique) for both the machines is calculated.

### B. At TPS commissioning

Beam data and all other dosimetric properties of both the machines are fed into the Eclipse treatment planning system (TPS) (Varian) version 11.0 in accordance with the vendor's specification and recommendations. Once the machine type is selected in Eclipse, it automatically takes all required basic machine characteristics from the available machine library data for beam modelling. Only measured PDDs, profiles, output factors, and absolute dose calibration factor are fed for the generation of beam model. Measured DLG and MLC transmission values are added as add‐on dosimetric parameter to the machine. These values can be further tweaked if necessary to improve the test results of a series of prerequisite QA performance tests for TPS and machines. In our context only the accuracy of the dose‐calculation method of TPS is evaluated individually for both the machines as part of the TPS QA.^10^, ^11^ Accordingly, chamber measurements (CC01 chamber) at different specified locations in a water phantom for a set of field sizes (3×3,10×10, and $25\times 25\;{\text{cm}}^{2}$) are compared with the TPS‐calculated value in two different scenarios (jaws only and MLC only). In addition, IMRT commissioning tests are performed in the TPS for both the machines separately.^(12)^ A specific set of test plans as specified in TG119 are then delivered at the machines. These test shapes and plans are representative of common clinical treatments and are used to test the overall accuracy of our IMRT commissioned system. The results are evaluated using both chamber measurements and 2D planar dosimetry. The portal imager is used for measuring 2D planar image and the results are analyzed using Portal Dosimetry (Varian). Gamma criteria used for 2D planar analysis are 3 mm and 3% as distance‐to‐agreement (DTA) and dose difference (DD), respectively. The tolerance limit is area gamma <1 is greater than 95%.

It is vital to evaluate the accuracy of beam matching between the two machines, especially in advanced patient treatment techniques like IMRT and RapidArc, beforepronouncing the machines “dosimetrically equivalent.” For this purpose 10 patient plans belonging to each technique (IMRT and RapidArc) and treatment sites (H&N and pelvis) for a total of 40 patient plans which were earlier treated on the Clinac iX machine at our hospital are chosen. The treatment plans are migrated to the Varian 600 C/D (Unique) machine in the Eclipse TPS and the dose recalculated for the same field fluence, field size and MUs of the Clinac iX machine using the option of “calculate with fixed MU.” In both cases, analytical anisotropic algorithm (AAA) is used for dose calculation with a grid resolution kept at 2.5 mm. The plans are compared to find the dosimetric difference between the two machines. The dose‐volume histogram (DVH) of target volumes and critical organs are used for evaluation.

#### B.1 Head and neck (H&N)

The simultaneous integrated boost (SIB) mode of dose prescription is usually practiced in H&N tumors. Accordingly, the dose prescribed to PTV I (high‐risk), PTV II (intermediate‐risk), and PTV III (low‐risk) volumes are 69.3 Gy, 66 Gy, and 59.4 Gy over 33 fractions, respectively. Spinal cord and parotids are the major organs at risk (OARs). Clinically important dose parameters $D_{2},D_{5}\;D_{10},D_{50},D_{90},D_{95},D_{98}$ and $D_{\text{mean}}$ of dose‐volume histograms (DVHs) are compared and average variation is calculated for the PTVs and parotids between the two machine plans.^13^, ^14^ For serial organs like spinal cord $D_{2}\;D_{50}$ and $D_{\text{mean}}$ parameters are considered for comparison. Average variation of each volume at mentioned levels is analyzed.

##### B.1.1 Intensity‐modulated radiation therapy (IMRT)

Normally seven to nine fields of 6 MV X‐rays are used in IMRT planning. For inverse optimization, Dose Volume Optimizer (DVO) version 11.0.31 is employed in the Eclipse TPS. The average volume of PTV I, PTV II, PTV III, spinal cord, and left and right parotid are 100.5±48.4 cc,693.1±127.0 cc,214.8±88.2 cc,27.0±7.3cc, 27.9±7.4 cc, and 20.9±1.8 cc, respectively. The average monitor units for the plans are 1429.5±260.5 MU.

##### B.1.2 RapidArc

Planning of RapidArc usually involves two full arcs of 6 MV X‐rays. RapidArc plans are generated by the Progressive Resolution Optimizer (PRO) III version 11.0.31 working principle based on direct‐aperture optimization. The average volume of PTV I, PTV II, PTV III, spinal cord, and left and right parotid are 81.6±29.6 cc,673.0±166.4 cc,166.16±62.2 cc,24.5±5.1 cc,24.7±7.1 cc, and 23.0±3.8 cc, respectively. The average monitor units for the plans are 582.7±66.9 MU.

#### B.2 Pelvis

In case of pelvic tumors, a dose of 50 Gy in 25 fractions is usually prescribed to the PTV. Normally, organs like rectum, bladder, and femoral heads are the OARs involved in the treatment of pelvic tumors. Dose of $D_{2},D_{5},D_{10},D_{50},D_{90},D_{95},D_{98}$, and $D_{\text{mean}}$ parameter values are extracted from the PTV and OARs DVH, for the comparison between the two machines.^12^, ^13^ Average variation of each volume at mentioned levels is analyzed.

##### B.2.1 Intensity‐modulated radiation therapy (IMRT)

IMRT planning at pelvis involves the use of seven to nine fields of 6 MV X‐rays similar to the planning of H&N IMRT. The average volume of PTV, rectum, bladder, femoral head, and left and right parotid are 822.2±328.0 cc,82.1±25.8 cc,167.9±27.5 cc,66.5±27.5 cc and 66.2±26.6 cc, respectively. The average monitor units for the plans are 1375.8±103.5 MU.

##### B.2.2 RapidArc

Two conventional full arcs of 6 MV X‐rays are used for planning. The average volume of PTV, rectum, bladder, femoral head, and left and right parotid are 710.1±60.7 cc,68.8±21.0 cc,118.9±81.1 cc,55.5±8.2 cc, and 58.0±7.8 cc, respectively. The average monitor units for the plans are 710.1±60.7 MU.

## III. RESULTS AND DISCUSSION

### A. At machine commissioning

The PDD values of both the machines at different depths for different fields are shown in Table 1. For field sizes $8\times 8\;{\text{cm}}^{2}$ and above, at all depths (excluding the buildup region), the absolute percent difference of PDD comparisons made between the two machines are well within 1%. In the same region, the degree of absolute percent difference increases for field sizes less than $8\times 8\;{\text{cm}}^{2}$ and reaches a maximum deviation of 2.05% at the depth of 16.5 cm for $6\times 6\;{\text{cm}}^{2}$ field size. On comparing the buildup region doses between the two machines, the absolute percent difference varies considerably for all field sizes. A maximum of −2.39% deviation is seen at the field sizes of $6\times 6\;{\text{cm}}^{2}$ and $15\times 15\;{\text{cm}}^{2}$. Excluding the buildup region, PDD comparison between the two machine fetches an overall percent difference value less than 2.05%. PDD comparison between the two machines at field sizes $10\times 10\;{\text{cm}}^{2}$ and $20\times 20\;{\text{cm}}^{2}$ are shown in 1, 2, respectively.

**Table 1:** PDD at different depths and field sizes for Clinac iX and Unique.

		*Field Size*			
*Depth*	*Machine*	$4\times 4\;{\text{cm}}^{2}$	$10\times 10\;{\text{cm}}^{2}$	$20\times 20\;{\text{cm}}^{2}$	$40\times 40\;{\text{cm}}^{2}$
Dmax (cm)	Clinac iX	1.66	1.59	1.46	1.34
	Unique	1.50	1.50	1.35	1.23
${\text{PDD}}_{5}\%$	Clinac iX	84.4	86.6	87.8	88.5
	Unique	83.4	86.5	87.6	87.9
${\text{PDD}}_{10}\%$	Clinac iX	62.4	66.9	70.1	72.2
	Unique	61.2	66.9	70.0	71.9
${\text{PDD}}_{20}\%$	Clinac iX	33.8	38.5	42.9	46.1
	Unique	32.3	38.4	42.8	46.2

**Figure 1:**
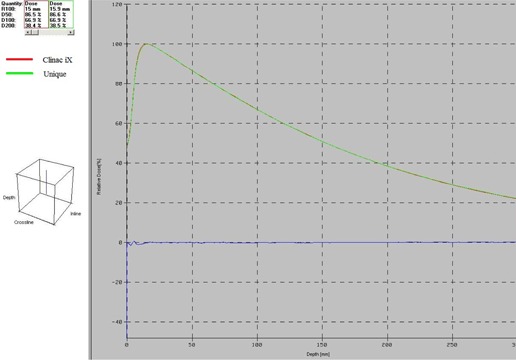
PDD comparison of Clinac iX and Unique machines for a field size of $10\times 10\;{\text{cm}}^{2}$.

**Figure 2:**
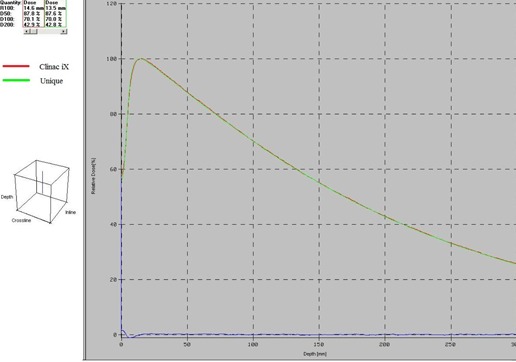
PDD comparison of Clinac iX and Unique machines for a field size of $20\times 20\;{\text{cm}}^{2}$.

Profile penumbra and field width measured at different depths are listed in Table 2 for both the machines. Profile disagreement within central 80% of the plane for all field sizes at all five depths between the two machines varies from −2.0% to 1.8%. The maximum variation −2% is found at the depth of 30 cm for the field sizes of $10\times 10\;{\text{cm}}^{2}$. The average absolute percent disagreement in the central 80% is 1.08±0.39%. The absolute percent difference in penumbra region for all the field sizes at all five depths between two machines varies from 4.4% to 20.7%. The maximum variation of 20.7% is found at the depth of 30 cm for the field sizes of $15\times 15\;{\text{cm}}^{2}$. The average disagreement in penumbra region for all the field sizes at all the depths is 10.6±4.9%.

TPR 20/10 values of 6 MV X‐rays are 0.667 and 0.670 for Clinac iX and Unique machines, respectively. Variation of TPR 20/10 value between the two machines is 0.45%. The output factors and their individual component values are tabulated in Table 3 for both the machines at different field sizes. For overall output factors, percentage variation between the two machines is in the range of 1.66% to −2.78% as the field size increases from $3\times 3\;{\text{cm}}^{2}$ to $40\times 40\;{\text{cm}}^{2}$. The phantom scatter factor remains almost constant with a maximum difference of 0.57%. The difference in head scatter factor of the two machines varies from 1.55% to −2.42% as the field size increases from $3\times 3\;{\text{cm}}^{2}$ to $40\times 40\;{\text{cm}}^{2}$. Since both the machines have different head characteristics and wave‐guide design the variations observed in output factors, especially the head scatter factors are quite understandable. The MLC transmission measured is 1.47% and 1.35% for Clinac iX and Unique machine respectively. The measured DLG value is same for both the machines and it is 2 mm. The percentage difference in MLC transmission between the two machines is 0.12%.

**Table 2:** Profile comparison for Clinac iX and Unique at different field sizes.

		$3\times 3\;{\text{cm}}^{2}$		$10\times 10\;{\text{cm}}^{2}$		$20\times 20\;{\text{cm}}^{2}$		$30\times 30\;{\text{cm}}^{2}$	
*Depth*	*Machine*	*Penumbra (mm)*	*Field Width (cm)*	*Penumbra (mm)*	*Field Width (cm)*	*Penumbra (mm)*	*Field Width (cm)*	*Penumbra (mm)*	*Field Width (cm)*
$D_{\text{max}}$	Clinac iX	4.9	3.06	5.3	10.25	5.5	20.48	5.5	30.68
	Unique	4.4	2.98	5.4	10.14	5.6	20.34	5.6	30.54
10 cm	Clinac iX	5.4	3.33	6.7	11.11	7.9	22.15	8.7	33.17
	Unique	4.9	3.27	6.8	11.00	7.8	22.03	8.7	33.04
20 cm	Clinac iX	5.7	3.64	8.2	12.13	13.0	24.16	17.3	36.14
	Unique	5.3	3.59	8.5	12.01	12.6	24.04	16.4	36.04

**Table 3:** Comparison of output factor, head scatter and phantom scatter factor for Clinac iX and Unique machine at different field sizes.

		*Field Size*			
	*Machine*	$3\times 3\;{\text{cm}}^{2}$	$10\times 10\;{\text{cm}}^{2}$	$20\times 20\;{\text{cm}}^{2}$	$40\times 40\;{\text{cm}}^{2}$
Output factor	Clinac iX	0.8666	1.000	1.0651	1.1263
	Unique	0.8810	1.000	1.0567	1.0950
**Head scatter factor**	Clinac iX	0.9134	1.000	1.0320	1.0606
	Unique	0.9275	1.000	1.0235	1.0347
Phantom scatter factor	Clinac iX	0.9488	1.000	1.0320	1.0620
	Unique	0.9499	1.000	1.0324	1.0583

### B. At TPS commissioning

The results of the TPS QA tests done as per TG53^(10)^ for both the machines are shown in 4, 5. The results are within the acceptable criteria for both machines. In IMRT commissioning tests show both machines can easily achieve the planning constraints as stated in TG119. From the results tabulated in 6, 7 it is seen that the machines can successfully deliver the plans within the specified tolerance criteria for all the shapes.

**Table 4:** Point‐dose results in different regions of a phantom for both machine at different field sizes (jaws only), per TG53.

		*Point Dose (cGy)*								
		*FS* $3\times 3\;{\text{cm}}^{2}$			*FS* $10\times 10\;{\text{cm}}^{2}$			*FS* $25\times 25\;{\text{cm}}^{2}$		
*Region of Measurement*	*Machine*	*Measured*	*Planned*	*% Variation*	*Measured*	*Planned*	*% Variation*	*Measured*	*Planned*	*% Variation*
Inner	Clinac iX	82.4	82.7	−0.36	95.6	95.3	0.31	104.6	103.6	0.97
	Unique	83.7	83.6	0.12	95.4	95.0	0.42	103.5	102.0	1.47
Outer	Clinac iX	1.2	0.8	0.48	4.1	3.9	0.21	7.6	7.9	−0.29
	Unique	1.4	0.8	0.72	3.8	3.5	0.32	7.7	7.0	0.69
Buildup	Clinac iX	95.5	96.8	−1.57	109.1	107.1	2.10	115.8	114.9	0.87
	Unique	99.2	99.2	0.00	109.2	107.8	1.47	116.8	114.1	2.65
Penumbra	Clinac iX	36.1	42.3	−7.50	44.1	50.1	−6.30	50.8	55.5	−4.54
	Unique	34.4	42.7	−9.93	37.8	49.9	−12.74	48.0	54.5	−6.38

**Table 5:** Point‐dose results in different regions of a phantom for both machine at different field sizes (MLC only) as per TG53.

		*Point Dose (cGy)*								
*Region of Measurement*		*FS* $3\times 3\;{\text{cm}}^{2}$			*FS* $10\times 10\;{\text{cm}}^{2}$			*FS* $25\times 25\;{\text{cm}}^{2}$		
	*Machine*	*Measure*	*d Planned*	*% Variation*	*Measured*	*Planned*	*% Variation*	*Measured*	*Planned*	*% Variation*
Inner	Clinac iX	82.8	82.7	0.12	95.7	95.3	0.42	103.9	103.6	0.29
	Unique	83.0	83.5	−0.60	94.9	95.0	−0.11	103.0	102.0	0.98
Outer	Clinac iX	1.5	0.8	0.85	4.2	3.9	0.31	8.0	7.9	0.10
	Unique	1.0	0.7	0.36	3.9	3.5	0.42	7.2	7.0	0.20
Build Up	Clinac iX Unique	98.4	96.8	1.93	107.6	107.1	0.52	116.5	114.9	1.54
	Unique	99.4	99.1	0.36	109.2	107.8	1.47	117.0	114.1	2.84
Penumbra	Clinac iX Unique	37.2	42.3	−6.17	41.5	50.1	−9.0	52.6	55.5	−2.80
	Unique	34.0	42.6	−10.30	37.2	49.9	−13.37	47.8	54.5	−6.57

**Table 6:** Gamma analysis and point‐dose results of IMRT plans of TG119 test cases in Clinac iX and Unique machines with confidence limit.

		*Clinac iX*				*Unique*			
		*Point Dose (Gy)*			*Planar Dosimetry*	*Point Dose (Gy)*			*Planar Dosimetry*
*Test Shape*	*Location*	*Measured*	*Planned*	*Deviation*	*Gamma* <1(%)	*Measured*	Planned	*Deviation*	*Gamma* <1(%)
Prostate	Isocenter 2.5 cm posterior	207.7	204.0	0.0185	99.89	205.5	204.6	0.0045	99.83
		127.2	128.1	−0.0045		127.0	128.4	−0.0070	
Head & Neck	Isocenter 4.0 cm posterior	209.69	205.9	0.0190	98.98	203.5	204.6	−0.0055	96.97
		115.5	118.7	−0.0160		110.9	118.0	−0.0355	
C‐Shape(E)	Isocenter 2.5 cm anterior	77.96	72.5	0.0273	98.23	71.3	69.9	0.0070	97.02
		221.86	218	0.0193		213.2	216.6	−0.0170	
C‐Shape(H)	Isocenter 2.5 cm anterior	48.27	46.1	0.0108	96.74	42.7	43.4	−0.0035	96.80
		221.56	218.7	0.0143		216	218.5	−0.0125	
Multi Target	Isocenter 4.0 cm superior	214.73	211.8	0.0146	99.36	211.2	211.7	−0.0025	98.87
		136.5	135.5	0.0050		136.1	136.4	−0.0015	
4.0 cm inferior	65.9	65.5	0.0020	64.2		65.6	−0.0070
Confidence Limits	High dose region		0.022		3.76		0.023		4.79
	Low dose region		0.033		3.76		0.036		

**Table 7:** Gamma analysis and point‐dose results of VMAT plans of TG119 test cases in Clinac iX and Unique machines with confidence limit.

		*Clinac iX*				*Unique*			
		*Point Dose (Gy)*			*Planar Dosimetry*	*Point Dose (Gy)*			*Planar Dosimetry*
*Test Shape*	*Location*	*Measured*	Planned	*Deviation*	*Gamma* <1(%)	*Measured*	Planned	*Deviation*	*Gamma* <1(%)
Prostate	Isocenter 2.5 cm posterior	200.7	200.2	0.0025	98.73	199.8	199.1	0.0035	99.40
		134.1	133.6	0.0025		133.0	132.8	0.0010	
Head & Neck	Isocenter 4.0 cm posterior	200.2	201.1	−0.0045	96.12	198.3	199.5	−0.0060	97.35
		139.7	138.3	0.0700		136.6	136.9	−0.0015	
C‐Shape(E)	Isocenter 2.5 cm anterior	60.7	60.2	0.0025	96.27	56.9	57.7	−0.0030	96.70
		208.3	209.1	−0.0040		203.8	206.9	−0.0155	
C‐Shape(H)	Isocenter 2.5 cm anterior	43.7	42.6	0.0055	94.39	39.1	39.9	−0.0040	97.61
		210.8	209.9	0.0045		204.1	207.9	−0.0190	
Multi Target	Isocenter 4.0 cm superior 4.0 cm inferior	210.1	209.4	0.0035	98.84	209.7	208.0	0.0085	96.20
		112.8	112	0.0040		110.6	109.5	0.0055	
		60.8	61.8	−0.0050		58.8	59.4	−0.0030	
Confidence Limits	High dose region		0.009		6.85		0.029		4.94
	Low dose region		0.011				0.008		

#### B.1 H&N

##### B.1.1 Intensity‐modulated radiation therapy (IMRT)

The average dose for different volumes of all organs for both machines for the same MU and fluence are tabulated in the Table 8, along with the dose difference between the two machines. With respect to PTV, at all dose levels, the average dose difference varies from 0.04 Gy to 0.40 Gy. The average dose difference for spinal cord at different dose levels $D_{2},D_{50},D_{\text{mean}}$ between the two machines varies from 0.51 Gy to 0.61 Gy. A variation of 0.02 Gy to 0.87 Gy is observed in the dose parameters for both the parotids. The dose variation is found to increase constantly across the parotid region as we move away from the PTVs. A maximum variation of 0.87 Gy is observed between the two machines at $D_{90}$ level for the right parotid.

**Table 8:** Average dose obtained from TPS for the same fluence and MU for the case of IMRT H&N (in Gy).

		$D_{2}$	$D_{5}$	$D_{10}$	$D_{50}$	$D_{90}$	$D_{98}$	$D_{\text{mean}}$
PTVI	Clinac iX	71.2±0.68	71.1±0.59	70.8±0.56	69.8±0.58	68.5±0.57	67.5±0.72	69.7±0.54
	Unique	71.0±0.65	70.7±0.61	70.4±0.58	69.4±0.61	68.2±0.55	67.3±0.64	69.3±0.57
	Difference	0.23±0.23	0.40±0.16	0.40±0.16	0.38±0.14	0.35±0.15	0.18±0.16	0.38±0.14
PTVII	Clinac iX	70.5±0.81	70.2±0.80	69.6±0.87	66.9±1.01	64.8±0.99	62.8±0.87	67.0±0.87
	Unique	70.3±0.77	69.8±0.81	69.2±0.88	66.5±1.02	64.5±1.00	62.7±0.84	66.7±0.90
	Difference	0.21±0.19	0.38±0.13	0.39±0.12	0.37±0.07	0.27±0.08	0.07±0.08	0.33±0.07
PTVIII	Clinac iX	63.7±1.16	62.3±0.96	61.1±0.90	59.2±0.65	57.8±0.85	56.1±1.02	59.4±0.67
	Unique	63.6±1.09	62.1±0.98	60.9±0.93	59.1±0.70	57.7±0.92	56.0±1.10	59.3±0.71
	Difference	0.04±0.12	0.20±0.1	0.18±0.10	0.12±0.11	0.05±0.11	0.07±0.23	0.12±0.11
Spinal	Clinac iX	39.7±1.21	–	–	35.1±1.47	–	–	41.9±1.48
	Unique	39.1±1.18	–	–	34.5±1.53	–	–	41.3±1.47
	Difference	0.50±0.15	–	–	0.60±0.13	–	–	0.61±0.23
Parotid L	Clinac iX	65.1±2.89	62.9±3.90	59.1±6.26	24.7±10.7	11.2±2.75	9.6±1.92	30.5±6.83
	Unique	65.0±2.87	62.6±3.89	58.8±6.34	23.9±10.8	10.3±2.75	8.9±1.91	29.9±6.85
	Difference	0.13±0.12	0.28±0.10	0.33±0.14	0.78±0.18	0.86±0.10	0.71±0.31	0.60±0.20
Parotid R	Clinac iX	64.0±2.47	61.3±2.86	57.5±3.61	23.3±5.00	11.3±1.96	9.7±1.64	29.4±3.12
	Unique	64.0±2.46	61.1±2.85	57.2±3.60	22.5±5.04	10.4±1.92	8.9±1.56	28.7±3.13
	Difference	0.02±0.15	0.21±0.08	0.26±0.09	0.81±0.09	0.87±0.09	0.82±0.10	0.68±0.05

##### B.1.2 RapidArc

In the H&N RapidArc plans, the average dose for different volumes of all organs for both machines for the same MU and fluence are shown in the Table 9, along with the difference in dose between the two machines.

In all PTVs, the average dose difference at all levels of dose $D_{2},D_{5},D_{10},D_{50},D_{90},D_{95},D_{98}$; $D_{\text{mean}}$ varies from 0.23 Gy to 0.82 Gy. The average dose difference between the two machines for spinal cord at different dose levels of $D_{2},D_{50}$, and $D_{\text{mean}}$ varies from 0.62 Gy to 0.72 Gy. In both parotids, an average dose variation of 0.38 Gy to 0.76 Gy is observed between the two machines at all levels of dose $D_{2},D_{5},D_{10},D_{50},D_{90},D_{95},D_{98}$, and $D_{\text{mean}}$. A maximum variation of 0.82 Gy is found between the two machines at $D_{98}$ level for PTV I.

**Table 9:** Average dose obtained from TPS for the same fluence and MU for the case of Rapid Arc H&N (in Gy).

		$D_{2}$	$D_{5}$	$D_{10}$	$D_{50}$	$D_{90}$	$D_{98}$	$D_{\text{mean}}$
PTVI	Clinac iX	71.8±0.53	71.5±0.51	71.2±0.49	70.1±0.41	68.8±0.45	67.9±0.52	70.1±0.42
	Unique	71.3±0.52	71.0±0.50	70.6±0.49	69.5±0.40	68.0±0.48	67.1±0.54	69.4±0.42
	Difference	0.55±0.15	0.56±0.15	0.60±0.15	0.67±0.12	0.78±0.15	0.82±0.19	0.68±0.13
PTVII	Clinac iX	71.1±0.52	70.5±0.51	69.9±0.50	67.5±0.56	65.2±0.70	62.9±0.79	67.5±0.54
	Unique	70.5±0.51	69.9±0.50	69.3±0.49	67.0±0.54	64.7±0.66	62.5±0.74	67.0±0.52
	Difference	0.57±0.13	0.59±0.11	0.58±.09	0.51±0.07	0.51±0.11	0.43±0.10	0.53±0.08
PTVIII	Clinac iX	64.5±1.48	62.8±0.94	61.6±0.61	60.1±0.51	58.5±0.57	56.9±0.72	60.1±0.51
	Unique	64.1±1.48	62.5±0.98	61.4±0.66	59.8±0.53	58.2±0.55	56.7±0.69	59.9±0.53
	Difference	0.32±0.12	0.31±0.13	0.25±0.10	0.26±0.07	0.27±0.08	0.23±0.07	0.26±0.08
Spinal	Clinac iX	37.5±1.33	–	–	33.6±1.40	–	–	38.7±3.14
	Unique	36.8±1.33	–	–	32.9±1.40	–	–	38.0±3.08
	Difference	0.72±0.06	–	–	0.62±0.08	–	–	0.67±0.11
Parotid L	Clinac iX	62.5±4.17	58.7±5.89	52.9±8.49	21.9±4.34	12.1±2.64	10.1±2.16	27.6±4.14
	Unique	62.1±4.21	58.2±5.98	52.4±8.61	21.2±4.35	11.4±2.64	9.5±2.18	27.0±4.16
	Difference	0.39±0.11	0.45±0.16	0.51±0.15	0.76±0.09	0.65±0.08	0.58±0.10	0.66±0.08
Parotid R	Clinac iX	63.2±5.13	59.8±7.31	54.5±9.81	21.7±4.11	11.7±2.00	9.7±1.85	28.1±4.04
	Unique	62.9±5.25	59.4±7.44	54.4±9.98	20.9±4.17	11.1±1.97	9.1±1.81	27.6±3.86
	Difference	0.38±0.17	0.42±0.16	0.44±0.19	0.76±0.09	0.65±0.10	0.58±0.12	0.49±0.44

Though there is not much difference in the overall dose variation it is clearly observable that variation in RapidArc plans are more than in IMRT plans for all the volumes. Among the volumes, PTV III has the least variation; 0.04 Gy to 0.32 Gy in both techniques. This is due to the presence of less critical organs in its vicinity. Interestingly the parotids variation as observed in IMRT tends to increase from $D_{2}$ to $D_{90}$ whereas in RapidArc the maximum variation is observed at $D_{50}$. The observed difference in parotid is due to the fact that the structure is overlapping with the PTV and the IMRT fields are delivered at fixed gantry angles. Variation in PTV I, PTV II, and spinal cord do not follow any specific trends.

#### B.2 Pelvis

##### B.2.1 Intensity‐modulated radiation therapy (IMRT)

The average dose for different volumes of all organs for both machines for the same MU and fluence are tabulated in the Table 10, along with the difference in dose between the two machines.

At different levels of dose $D_{2},D_{5},D_{10},D_{50},D_{90},D_{95},D_{98},D_{\text{mean}}$ the average dose difference for PTV varies from 0.29 Gy to 0.36 Gy. The average dose difference between the two machines for the structures adjacent to the target, such as the bladder and rectum, at different dose levels of $D_{2},D_{5},D_{10},D_{50},D_{90},D_{95},D_{98},D_{\text{mean}}$ varies from 0.33 Gy to 0.55 Gy. For less critical structures like femoral heads, a variation of 0.33 Gy to 0.42 Gy is observed at all levels of dose parameters. The maximum variation of 0.55 Gy was found between the two machines at rectum in $D_{90}$ levels.

**Table 10:** Average dose obtained from TPS for the same fluence and MU for the case of IMRT pelvis (in Gy).

		$D_{2}$	$D_{5}$	$D_{10}$	$D_{50}$		$D_{98}$	$D_{\text{mean}}$
PTV	Clinac iX	52.0±0.46	51.9±0.43	51.3±0.44	50.3±0.41	49.3±0.42	47.8±0.78	50.2±0.43
	Unique	51.6±0.47	51.2±0.48	50.9±0.48	50.0±0.41	48.9±0.43	47.5±0.75	49.9±0.43
	Unique	0.36±0.12	0.34±0.14	0.34±0.13	0.31±0.08	0.34±0.11	0.29±0.15	0.31±0.09
Rectum	Clinac iX	50.6±0.63	50.0±0.66	49.2±0.90	37.1±4.19	17.2±4.65	10.5±3.20	35.1±2.46
	Unique	50.1±0.62	49.5±0.62	48.7±0.84	36.6±4.22	16.7±4.65	9.98±3.04	34.7±2.47
	Difference	0.42±0.14	0.45±0.19	0.45±0.17	0.43±0.13	0.55±0.10	0.50±0.18	0.46±0.11
Bladder	Clinac iX	51.6±0.67	51.2±0.56	50.9±0.54	47.7±3.46	33.6±12.5	28.5±15.8	44.85±4.59
	Unique	51.2±0.62	50.8±0.55	50.5±0.54	47.2±3.38	33.2±12.5	28.2±15.7	44.4±4.54
	Difference	0.39±0.14	0.38±0.07	0.39±0.07	0.44±0.11	0.40±0.10	0.34±0.16	0.43±0.08
Lt.femur	Clinac iX	35.3±3.53	32.0±3.30	29.0±2.41	20.0±2.23	13.1±2.90	10.0±2.86	20.7±1.97
	Unique	35.0±3.53	31.6±3.32	28.6±2.43	19.7±2.24	12.7±2.91	9.6±2.85	20.3±1.98
	Difference	0.35±0.10	0.39±0.08	0.40±0.10	0.36±0.10	0.37±0.07	0.36±0.09	0.37±0.07
Rt.femur	Clinac iX	35.3±4.22	32.4±4.16	29.5±3.61	20.3±2.75	12.8±2.50	9.8±2.89	21.0±2.58
	Unique	34.9±4.24	32.0±4.16	29.1±3.60	19.9±2.74	12.5±2.47	9.5±2.85	20.6±2.58
Difference	0.40±0.09	0.42±0.09	0.41±0.08	0.39±0.09	0.38±0.07	0.33±0.10	0.39±0.07

##### B.2.2 RapidArc

In pelvis RapidArc plans, the average dose for different volumes of all organs for both machines for the same MU and fluence are shown in the Table 11, along with the dose difference between the two machines. Average dose difference for PTV between the two machines at different levels of dose $D_{2},D_{5},D_{10},D_{50},D_{90},D_{95},D_{98},D_{\text{mean}}$ varies from 0.29 Gy to 0.34 Gy. Looking at the statistics of critical structures such as the bladder and rectum at different dose levels of D2, $D_{5},D_{10},D_{50},D_{90},D_{95},D_{98},D_{\text{mean}}$, the deviation is found to vary from 0.30 Gy to 0.53 Gy. In case of femoral heads the average dose difference between the two machines at different levels of dose varies from 0.21 Gy to 0.42 Gy. The overall maximum variation of 0.53 Gy between the two machines was found for bladder at $D_{50}$ and $D_{5}$ levels.

**Table 11:** Average dose obtained from TPS for the same fluence and MU for the case of Rapid Arc pelvis (in Gy).

		$D_{2}$	$D_{5}$	$D_{10}$	$D_{50}$	$D_{90}$	$D_{98}$	$D_{\text{mean}}$
PTV	Clinac iX	52.1±0.45	51.9±0.44	51.6±0.43	50.7±0.35	49.6±0.33	48.4±0.46	50.6±0.35
	Unique	51.9±0.43	51.6±0.42	51.3±0.42	50.4±0.38	49.3±0.34	48.0±0.45	50.3±0.36
	Difference	0.29±0.09	0.29±0.10	0.30±0.11	0.34±0.09	0.33±0.14	0.34±0.11	0.34±0.09
Rectum	Clinac iX	51.0±0.73	50.3±1.07	49.2±1.70	27.8±6.51	11.9±2.60	9.05±2.04	29.5±3.55
	Unique	50.6±0.70	50.0±1.04	48.9±1.68	27.3±6.51	11.5±2.56	8.75±2.03	29.1±3.51
	Difference	0.32±0.10	0.33±0.09	0.35±0.08	0.46±0.20	0.40±0.17	0.30±0.18	0.41±0.15
Bladder	Clinac iX	51.7±0.53	51.3±0.61	50.7±0.97	44.1±5.90	32.3±7.17	25.3±9.72	42.7±4.75
	Unique	51.2±0.65	50.7±0.69	50.2±1.03	43.5±5.90	31.9±7.18	24.9±9.64	42.3±4.75
	Difference	0.52±0.18	0.53±0.15	0.52±0.13	0.53±0.19	0.43±0.11	0.37±0.17	0.47±0.11
Lt.femur	Clinac iX	37.2±2.71	34.1±2.31	31.1±1.91	21.8±2.00	16.6±2.23	14.8±2.42	22.9±2.00
	Unique	36.8±2.68	33.7±2.25	30.7±1.86	21.5±1.94	16.3±2.19	14.6±2.34	22.6±1.94
	Difference	0.41±0.16	0.41±0.14	0.38±0.11	0.35±0.14	0.26±0.12	0.21±0.14	0.35±0.13
Rt.femur	Clinac iX	35.8±4.21	33.1±3.53	30.4±2.94	21.9±2.80	16.2±2.52	14.5±2.57	22.7±2.60
	Unique	35.4±4.23	32.6±3.48	29.9±2.90	21.5±2.76	16.0±2.46	14.2±2.52	22.3±2.55
	Difference	0.40±0.16	0.42±0.15	0.41±0.15	0.34±0.13	0.28±0.13	0.27±0.13	0.41±0.25

Unlike H&N, in pelvic plans variations observed in the organs do not seem to be technique‐oriented. They are due to deep‐seated as well body‐centered PTV. The variations in PTV do not follow any trends. Maximum dose variation in rectum is observed at $D_{90}$ and $D_{50}$ for IMRT and RapidArc respectively. Surprisingly the variation pattern observed in rectum is similar to that of the parotids in H&N, rectum being an overlapping structure. The bladder shows slightly higher variation in the RapidArc plans. Femoral heads never follow any trends, neither in volumes nor in technique.

A closer look at beam‐matching results reveals that notable dose difference is observed in H&N patients, especially in RapidArc techniques. Generally, the complexity of H&N plans is relatively greater than in pelvis plans because more critical organs are involved in the H&N region. In addition, the results show that volumes receive more doses in Clinac iX machine as compared to 600 C/D (Unique) machine for the same monitor units. This is due to the reciprocal effect of output factor variation observed at particular field sizes between the machines, which in turn is due to the different head designs employed between the two linear accelerators.

## V. CONCLUSION

The accuracy of “beam matching” between the two machines for 6 MV X‐rays when the linear accelerators are set within the manufacturer's specifications was evaluated in a systematic and detailed manner. The results of comparisons made between the beam‐matched machines for PDDs, profiles, and output factors are within the range of satisfaction. Comparative dosimetric analysis of IMRT and RapicArc patient plans swapped between the two machines at the TPS level in both the H&N and pelvis treatment sites are well within clinically acceptable tolerance. Overall results show that in a busy center, during down times, patients can be shifted across the beam‐matched machines with the assurance of pretreatment verification alone, without the need for replanning.

## COPYRIGHT

This work is licensed under a Creative Commons Attribution 3.0 Unported License.

## Supporting information

Supplementary Material

Supplementary Material

Supplementary Material

Supplementary Material

Supplementary Material

Supplementary Material

Supplementary Material

Supplementary Material

Supplementary Material

Supplementary Material

Supplementary Material
