# Supplementary figures and images for: Is the dose distribution distorted in IMRT and RapidArc treatment when patient plans are swapped across beam‐matched machines?
Source: J Appl Clin Med Phys. 2016 Sep 8;17(5):111–23. doi: 10.1120/jacmp.v17i5.6104 (PMC5874098; doi:10.1120/jacmp.v17i5.6104)

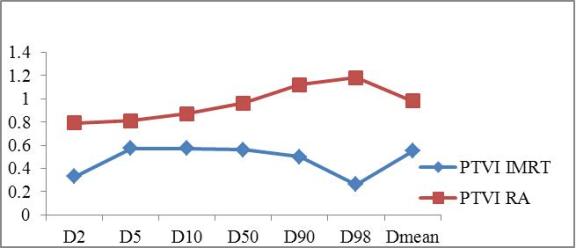

Supplement: Supplementary file 1 — Supplementary Material [file ACM2-17-111-s001.JPG]

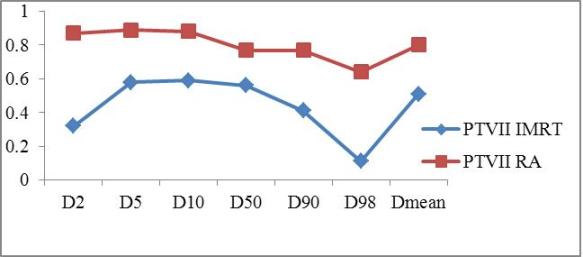

Supplement: Supplementary file 2 — Supplementary Material [file ACM2-17-111-s002.JPG]

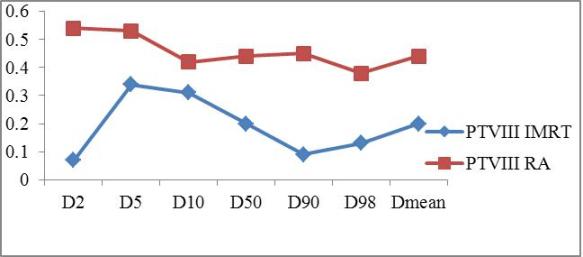

Supplement: Supplementary file 3 — Supplementary Material [file ACM2-17-111-s003.JPG]

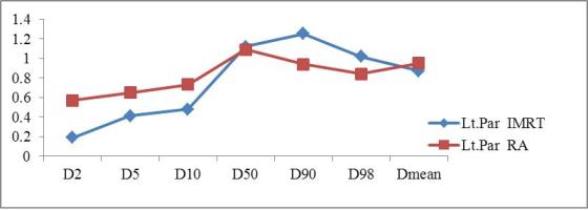

Supplement: Supplementary file 4 — Supplementary Material [file ACM2-17-111-s004.JPG]

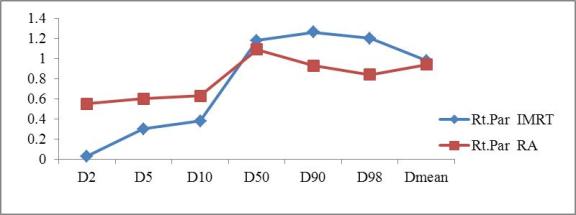

Supplement: Supplementary file 5 — Supplementary Material [file ACM2-17-111-s005.JPG]

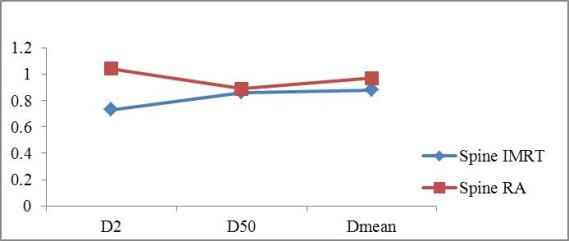

Supplement: Supplementary file 6 — Supplementary Material [file ACM2-17-111-s006.JPG]

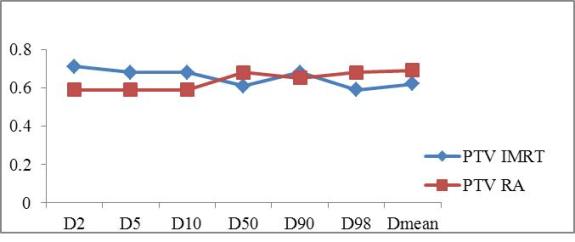

Supplement: Supplementary file 7 — Supplementary Material [file ACM2-17-111-s007.JPG]

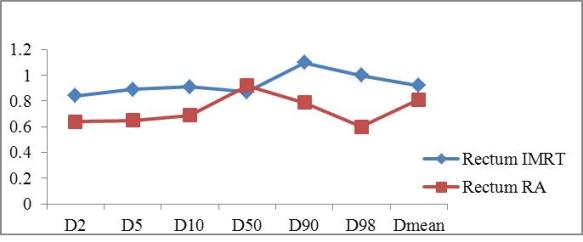

Supplement: Supplementary file 8 — Supplementary Material [file ACM2-17-111-s008.JPG]

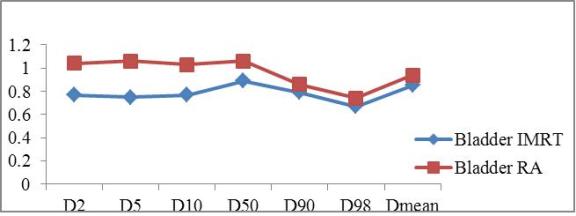

Supplement: Supplementary file 9 — Supplementary Material [file ACM2-17-111-s009.JPG]

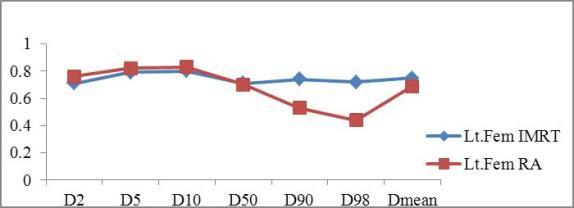

Supplement: Supplementary file 10 — Supplementary Material [file ACM2-17-111-s010.JPG]

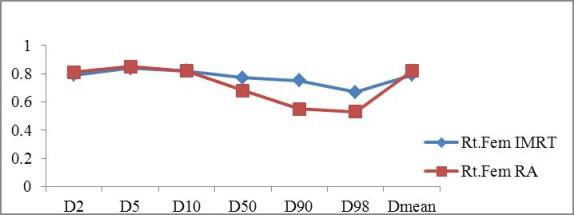

Supplement: Supplementary file 11 — Supplementary Material [file ACM2-17-111-s011.JPG]
